# Evaluation of the London Measure of Unplanned Pregnancy in a United States Population of Women

**DOI:** 10.1371/journal.pone.0035381

**Published:** 2012-04-19

**Authors:** Diane Morof, Jody Steinauer, Sadia Haider, Sonia Liu, Philip Darney, Geraldine Barrett

**Affiliations:** 1 Department of Obstetrics, Gynecology and Reproductive Sciences, San Francisco General Hospital and the Bixby Center for Global Reproductive Health, University of California San Francisco, San Francisco, California, United States of America; 2 Department of Obstetrics and Gynecology, Beth Israel Deaconess Medical Center, Boston, Massachusetts, United States of America; 3 Department of Family Medicine, University of California Davis, Davis, California, United States of America; 4 Health Sciences and Social Care, Brunel University, West London, United Kingdom; RAND Corporation, United States of America

## Abstract

**Objective:**

To evaluate the reliability and validity of the London Measure of Unplanned Pregnancy (a U.K.-developed measure of pregnancy intention), in English and Spanish translation, in a U.S. population of women.

**Methods:**

A psychometric evaluation study of the London Measure of Unplanned Pregnancy (LMUP), a six-item, self-completion paper measure was conducted with 346 women aged 15–45 who presented to San Francisco General Hospital for termination of pregnancy or antenatal care. Analyses of the two language versions were carried out separately. Reliability (internal consistency) was assessed using Cronbach's alpha and item-total correlations. Test-retest reliability (stability) was assessed using weighted Kappa. Construct validity was assessed using principal components analysis and hypothesis testing.

**Results:**

Psychometric testing demonstrated that the LMUP was reliable and valid in both U.S. English (alpha = 0.78, all item-total correlations >0.20, weighted Kappa = 0.72, unidimensionality confirmed, hypotheses met) and Spanish translation (alpha = 0.84, all item-total correlations >0.20, weighted Kappa = 0.77, unidimensionality confirmed, hypotheses met).

**Conclusion:**

The LMUP was reliable and valid in U.S. English and Spanish translation and therefore may now be used with U.S. women.

## Introduction

Approximately half of all pregnancies in the U.S. are considered to be unintended [1] and a long standing aim of U.S. public health policy has been to reduce the number of unintended pregnancies [2], [3]. Hence, the monitoring of pregnancy intention status of pregnancies that have occurred, via national and sub-national surveys, has been carried out for more than 50 years. The most influential survey in the U.S. and the source of national statistics about unplanned pregnancy is the federally-sponsored National Survey of Family Growth (NSFG). Despite the well-established nature of the NSFG questions to assess unplanned pregnancy, there has been a growing awareness of the limitations of these (and similar) questions, exposing a need for a more accurate measure of pregnancy intendedness, in particular a measurement method that can tap into more nuanced feelings and behaviour in relation to conception [4], [5], [6], [7], [8], [9], [10], [11], [12], [13]. The London Measure of Unplanned Pregnancy (LMUP), which was developed in the U.K., can potentially address this need. It is a new measure of pregnancy intention/planning with excellent psychometric properties [14], [15]. The measure does not assume that women have fully formed childbearing plans, that women's intentions are necessarily congruent with their actions, or that women are universally rational and see fertility as within their control. The measure can be used with any pregnancy regardless of outcome. The LMUP is self-administered in English, and it comprises six questions (contraceptive use, timing, intention, desire for a baby, partner discussion, and pre-conceptual preparations) via which women report the circumstances of their current or recent pregnancy. Each item is scored 0–2, with women's total score ranging from 0 to 12. Each point increase represents an increase in pregnancy planning/intention, with the authors recommending a broad preliminary interpretation of scores of 0–3 as unplanned, 4–9 ambivalent, and 10–12 planned. These properties of the LMUP would make it a useful addition to the U.S. toolkit for studying pregnancy intention. In this study we evaluate the psychometric properties of the LMUP (in U.S. English and Spanish translation) in a U.S. population of women to assess its suitability for use in the U.S.

## Methods

### Ethics statement

IRB approval was granted for this study by the Committee on Human Research at University of California, San Francisco. Written informed consent was obtained for all study participants.

Paper questionnaires were prepared in English and Spanish. Each questionnaire contained the six items of the LMUP, plus socio-demographic questions. For the U.S. English version of the LMUP, no changes were made to the wording of the items however the instruction “please tick” was changed to “please select” throughout, in keeping with usual U.S. questionnaire wording. The translation of the LMUP into Spanish followed the standard procedure of translation and back-translation and was carried out by a professional translation company.

The U.S. English and Spanish versions of the questionnaire were pre-tested using brief cognitive (verbal probing) interviews [16]. Ten English-speaking and ten Spanish-speaking women were approached in antenatal and abortion clinics of San Francisco General Hospital. The aim of the interviews was to assess their understanding of the language and wording of the questionnaire and to gauge their opinions on its acceptability. The reading level of the U.S. English version of the LMUP was also assessed using the Flesch-Kincaid grade level scale.

A field test was carried out at the San Francisco General Hospital where the questionnaire was distributed to a total of 350 women: 150 in the abortion clinic (75 English and 75 Spanish-speaking) and 200 women in the antepartum clinic (100 English- and 100 Spanish-speaking). Women between 15 and 45 years of age were approached and those with basic literacy in English or Spanish were eligible to take part. The sample composition was designed to reflect the ratio of abortions to live births that is found in this low-income population [1] and to meet the sample size requirements for psychometric measure evaluation [17], [18]. All women were asked if they would consent to completing the questionnaire a second time. In the abortion clinic, women who consented were sent the questionnaire at least two weeks later (with follow-up reminders for non-responders). Women in the antepartum clinic were either sent a questionnaire two weeks later or were sent the questionnaire after their baby was born (with follow-up reminders for non-responders); in order to have equal numbers in these groups women were put into the ‘two-week’ or ‘postpartum’ category by the week they were seen in clinic. Logistic regression analysis was used to examine the differences between those returning a retest questionnaire and those not. For both the standard test-retest and the longer term post-partum test-retest, there were a number of late returners of the questionnaire; a decision was made to retain all women in the test-retest groups, regardless of time interval between completions, provided the women had valid scores (i.e. no more than 3 incomplete answers), and their pregnancy situations were appropriate to the analysis group. Where women had missing data for three items or fewer, total LMUP scores were calculated by imputing mean item values [14].

Acceptability of the LMUP was determined during the cognitive interviews. Rates of missing data in the field test were further assessed to give an indication of items that might have problems with acceptability or validity [19]. Item category endorsement values were examined to identify any category than had an endorsement frequency of ≥80%. The distributions of total scores were examined to ensure all parts of the scale were being used, as an indicator of appropriate targeting of the measure.

Reliability (internal consistency) was assessed using the Cronbach's alpha statistic [20] (>0.7 indicating acceptable reliability) and corrected item-total correlations (<0.2 indicating that the item is contributing little to the homogeneity of the scale) [18]. Test-retest reliability (stability) was examined in two ways: 1) a standard test-retest (aiming for at least a two week interval between completions); and 2) a longer term postpartum test-retest (with the birth of a baby between completions). The rationale for the latter test was the evidence that women's scores may be unstable over this transition [21]. In both instances, test-retest reliability was measured using the weighted *k* (the non-parametric equivalent of the intra-class correlation coefficient), with a score of 0.41–0.60 indicating moderate agreement, 0.61–0.80 substantial agreement, and 0.81–1.00 almost perfect agreement [22]. We also compared mean scores to assess the direction of any score change, and carried out a paired t-test to assess significance.

Construct validity was assessed by two methods: principal component analysis and hypothesis testing. We used principal component analysis (without rotation requesting as many factors as there were Eigenvalues >1) to test the hypothesis that all items would load onto one factor (i.e. measuring the same construct). For hypothesis testing we tested two hypotheses that were strongly supported by the U.S. literature [23], [24], [25], [26] and have been demonstrated previously with the LMUP [14], [27]: 1) that higher scores will be associated with pregnancies continued to term and lower scores with pregnancies ending in abortion; and 2) living with a married partner will be associated with higher scores than not living with a married partner. Mann-Whitney U tests were carried out to assess significance.

Finally, a simple exploratory analysis was carried out, based on the principles of modern test theory, as opposed to classical test theory, which informed the development of the original measure and above analyses. A Mokken scaling procedure (monotone homogeneity assumption) was carried out using Stata 9.0, examining the full dataset. Items with a Loevinger H coefficient >0.3 were eligible for scaling [28], [29]. (The Loevinger H coefficient relates to Guttman errors, with a lower H value indicating more observed Guttman errors.) The results of Mokken analysis allows investigators to see whether the items conform to a probalistic Guttman structure, i.e. that items vary in ‘difficulty’, some being easy to endorse, some being more difficult to endorse, and that respondents who have a particular level of the construct (in this case pregnancy planning/intention) should broadly endorse items up to the level of their construct and then not endorse items beyond that. The whole scale is also assessed by Loevinger H coefficient, with <0.4 meaning the scale is “weak”, 0.4 to 0.49 meaning the scale is “medium”, and ≥0.5 meaning the scale is “strong” [28]. The construction of an adequate scale confirms that the raw score can be used to order respondents on the construct being measured [29].

Analyses were carried out using SPSS for Windows 15 (SPSS Inc.: *SPSS for Windows*. Version 15 edition. Chicago, Illinois, USA; 2007) and Stata 9 (Stata Corp. 2005. Statistical Software: Release 9.0. College Station, TX: Stata Corporation).

## Results

### Samples

The pre-test sample comprised 20 women; ten English-speaking and ten Spanish-speaking; ten abortion patients and ten continuing pregnancies. The average age for the English-speaking women was 30 years and for the Spanish speakers was 32 years. Three hundred and forty-six women consented to take part in the main field test; 345 answered at least one item of the LMUP and the socio-demographic characteristics of these women are shown in Table 1. Two hundred and fourteen women (62.0%) returned a retest questionnaire; returners were, after adjustment, significantly more likely to have completed the U.S. English version of the LMUP, have fewer children, and be continuing their pregnancy to term (Table 2). Of the 214 women returning a retest questionnaire, 97.2% (208 total, 90 Spanish and 118 English) had valid scores for both the test and retest.

**Table 1:** Socio-demographic Characteristics of English and Spanish-speaking women taking part in the U.S. London Measure of Unplanned Pregnancy (LMUP) field test.

Socio-demographic Characteristic	U.S. English LMUP version completed n = 173	Spanish LMUP version completed n = 172
Age	***mean (SD)***	***mean (SD)***
	26.0 (6.5)	26.2 (5.8)
Pregnancy outcome	***n (%)***	***n (%)***
abortion	72 (41.6)	72 (41.9)
continue pregnancy	101 (58.4)	100 (58.1)
Children		
0	84 (48.6)	54 (33.1)
1	39 (31.3)	51 (31.3)
2	21 (12.1)	41 (25.2)
3	15 (8.7)	8 (4.9)
4+	14 (8.1)	9 (5.5)
Who women live with (excluding children)		
husband	30 (17.6)	49 (30.6)
partner	49 (28.8)	50 (31.3)
not husband/partner	91 (53.5)	61 (38.1)
Race/ethnicity		
White	25 (16.3)	0 (0.0)
African American	53 (34.6)	1 (0.6)
Asian	27 (17.6)	0 (0.0)
Latina	36 (23.5)	147 (91.9)
Other	12 (7.8)	12 (7.5)
Total household income		
less than $30,000	92 (53.2)	63 (36.6)
$30,000 to $60,000	25 (14.5)	8 (4.7)
more than $60,000	3 (1.7)	1 (0.6)
don't know/missing	53 (30.6)	100 (58.1)

**Table 2:** Characteristics of women returning retest questionnaire.

	% (n)	p	Adjusted odds ratio (95% CI)	p
Language version of questionnaire		0.005		0.006
Spanish	54.7 (94)		0.51 (0.31 to 0.83)	
U.S. English	69.4 (120)		1.0	
Pregnancy outcome		0.02		0.033
abortion	54.9 (79)		0.60 (0.37 to 0.96)	
continue pregnancy	67.2 (135)		1.0	
Age group		0.214	-	
<20	62.7 (32)			
20–24	62.3 (66)			
25–29	54.4 (49)			
30–34	61.7 (37)			
35–39	81.5 (22)			
40+	72.7 (8)			
Children		<0.001		<0.001
0	68.8 (95)		1.0	
1	73.3 (66)		1.48 (0.81 to 2.73)	
2	54.8 (34)		0.72 (0.38 to 1.37)	
3+	37.0 (17)		0.27 (0.13 to 0.56)	
Who women live with (excluding children)		0.073		
husband	67.1 (53)			
partner	70.7 (70)			
not husband/partner	57.2 (87)			
Total household income		0.68	-	
less than $30,000	63.9 (99)		-	-
$30,000+	64.9 (24)			
don't know/missing	59.5 (91)			

### Acceptability and targeting

Pre-testing showed the LMUP to be acceptable to both English and Spanish-speaking women, and no changes to the wording of either the U.S. English or Spanish LMUP items were made. The reading level of the LMUP was age 11 for the U.S. English version (Flesch-Kincaid grade 5.9).

There were extremely low levels of missing data with the U.S. English version of the LMUP, and low levels with the Spanish version (Table 3). No response category had an endorsement value ≥80%. The item with the least variability in endorsement was item 1 (contraception) with the majority of women (70.6% of U.S. English and 68.5% of Spanish version) scoring 2.

**Table 3:** Missing data with the U.S. English and Spanish versions of the London Measure of Unplanned Pregnancy.

LMUP version completed	Items	Missing data n (%)	Item total correlations	Cronbach's alpha	Component loadings
U.S. English				0.78	Eigenvalue = 2.9
	1 (contraception)	3 (1.7)	0.21		0.31
	2 (timing)	2 (1.2)	0.64		0.80
	3 (intention)	0 (0.0)	0.69		0.84
	4 (desire)	1 (0.6)	0.62		0.79
	5 (partner discussion)	5 (2.9)	0.62		0.78
	6 (preparations)	4 (2.3)	0.37		0.51
Spanish				0.84	Eigenvalue = 3.4
	1 (contraception)	10 (5.8)	0.22		0.30
	2 (timing)	8 (4.7)	0.65		0.78
	3 (intention)	3 (1.7)	0.83		0.91
	4 (desire)	6 (3.5)	0.78		0.88
	5 (partner discussion)	12 (7.0)	0.80		0.88
	6 (preparations)	10 (5.8)	0.43		0.56

**Figure 1:**
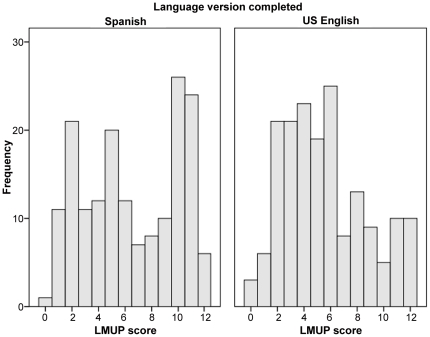
Distribution of English and Spanish London Measure of Unplanned Pregnancy scores in a U.S. population of women. Scores are presented for English (left) and Spanish (right) in Figure 1. All scores were represented in each language. A non-normal distribution was noted for each language.

All women answering the U.S. English version, and 169 (98.3%) answering the Spanish version, answered at least three LMUP items and were therefore eligible to have an LMUP total score. All scores were represented in both language versions (Figure 1). The distributions were non-normal, with 29.5% (51) of U.S. English completers and 26.0% (44) of Spanish completers scoring 0–3, 56.1% (97) and 40.8% (69) respectively scoring 4–9, and 14.5% (44) and 33.1% (56) respectively scoring 10–12.

### Reliability

The Cronbach alphas were above 0.7 for both versions and all item-total correlations were above 0.2 (Table 3).

For the standard test-retest, the median time between completion of the test and the retest questionnaire was 19 days (25^th^ and 75^th^ percentiles: 16, 31; range 371) for U.S. English completers and 22 days (25^th^ and 75^th^ percentiles: 15, 30, range 103) for Spanish completers. The weighted *k* was 0.72 for the U.S. English version and 0.77 for the Spanish version. Also, there was no significant change in group mean scores between administrations, with a mean of 5.0 (SD 3.1) at first administration and 5.0 (SD 3.1) at second administration for the U.S. English completers (p = 0.76), and a mean of 6.8 (SD 3.8) and 7.0 (SD 3.7) respectively for the Spanish completers (p = 0.36).

For the postpartum test-retest, the median time between completion of the questionnaires was 105 days (25^th^ and 75^th^ percentiles: 39, 166; range 524) for U.S. English completers and 127 days (25^th^ and 75^th^ percentiles: 50, 214, range 481) for Spanish completers. The weighted *k* was 0.55 for the U.S. English version and 0.55 for the Spanish version. The group mean LMUP scores did not change significantly between administrations, with a mean of 7.1 (SD 3.1) at first administration and 7.0 (SD 2.9) at second administration for the U.S. English completers (p = 0.73), and a mean of 9.1 (SD 2.2) and 9.3 (SD 2.0) respectively for the Spanish completers (p = 0.49).

### Validity

The results of principal components analysis confirmed that all variables loaded onto one component in both language versions, with all component loadings greater than 0.3 (Table 3). The results of hypothesis testing showed that both hypotheses were met for both language versions. For hypothesis one, that higher scores will be associated with pregnancies continued to term and lower scores with pregnancies ending in abortion, the median LMUP score for U.S. English completers continuing their pregnancy was 7 (25^th^ and 75^th^ percentiles: 5, 10; range 0–12) compared with a median of 3 (25^th^ and 75^th^ percentiles: 2, 5; range 0–9) for those opting for abortion (p<0.001), and the median LMUP score for Spanish completers continuing their pregnancy was 9.5 (25^th^ and 75^th^ percentiles: 6, 11; range 2–12) compared with a median of 3 (25^th^ and 75^th^ percentiles: 2, 5; range 0–12) for those opting for abortion (p<0.001). For hypothesis two, the median LMUP score for U.S. English completers living with a husband was 8 (25^th^ and 75^th^ percentiles: 5, 11; range 1–12) compared with a median of 5 (25^th^ and 75^th^ percentiles: 3, 7; range 0–12) for those not (p<0.001), and the median LMUP score for Spanish completers living with a husband was 10 (25^th^ and 75^th^ percentiles: 5.5, 11; range 1–12) compared with a median of 5 (25^th^ and 75^th^ percentiles: 3, 10; range 0–12) for those not (p = 0.02).

### Scaling

The Mokken analysis showed that items differed in their difficulty, with item 1 (contraceptive use) being easiest to endorse, followed by items 2, 4, 5, and 3, and item 6 (pre-conceptual preparations) as hardest to endorse. The items conformed to a basic Guttman structure (Loevinger H values: item 1, 0.25; item 2, 0.54; item 3, 0.66; item 4, 0.60; item 5, 0.59; item 6, 0.37). The Mokken scaling procedure selected five items into the scale (H = 0.60 for whole scale), as item 1 narrowly missed selection with a Loevinger H coefficient <0.3. However, even with item 1 included, the Loevinger H coefficient for the overall 6-item scale was still 0.53.

## Discussion

The LMUP versions in U.S. English and Spanish translation are valid and reliable according to internationally-accepted psychometric criteria in a U.S. population of English and Spanish speaking women. These LMUP versions can be used with confidence in research studies as a measure of unintended pregnancy in the U.S.

The study evaluated the LMUP in a low income population and this population may not reflect the women at risk of pregnancy in the U.S. population as a whole. However, low SES women in the U.S. have more limited access to pregnancy prevention methods and are at higher risk of undesired pregnancy and abortion, and it is therefore vitally important to confirm that the LMUP is valid for use among this group. The sample bias towards low income/low SES women may explain the low variability in endorsement in item 1 (contraceptive use). It is worth noting, however, that the study was conducted in X, which has exceptional resources available to assist low income women, including non-citizens, to prevent unplanned pregnancies [30], therefore it is less likely that low contraceptive use was simply due to lack of access to contraceptive services.

This evaluation of the LMUP meets internationally accepted standards for psychometric validation studies [31], [32], and is directly comparable with the original U.K. validation study [14], including the performance of a postpartum test-retest (which not a standard feature in psychometric studies for obvious reasons). The reliability coefficients (internal consistency/Cronbach's alpha, and standard test-retest) in this study are slightly lower (>0.7) than the U.K. development study (>0.9) but are entirely acceptable according to standard psychometric criteria, and appropriate for the population-level (as opposed to individual-level) use for which the LMUP is intended.

A strength of this study is that women with abortions were included in the standard test-retest, which was not possible for ethical reasons in the U.K. The U.S. LMUP study provides something new in this context, and it is reassuring that the inclusion of women with abortions did not seem to diminish the reliability of the LMUP measure.

The postpartum term test-retest results raise some questions. The reliability coefficients for both the U.S. English and Spanish versions are in Landis and Koch's “moderate” agreement banding. This is different to the U.K. study, where the postpartum test-retest weighted Kappa was >0.80. One point of reassurance though is that for neither language version was there an increase in scores at the group level (which is contrary to previous evidence by Joyce et al [21] but consistent with the U.K. LMUP findings). Our interpretation of this is that although there might be only moderate agreement of scores at the level of the individual woman, at the population level the scores seem to be stable, which means we can have confidence in the prevalence estimates produced among postpartum women.

In this study, Mokken analysis was carried out —no modern test theory analyses have been carried out on the U.K. data so far. The Mokken analysis indicated that item 1 (contraception) was not contributing greatly to the scale however it is not a critical problem as the scale was still strong with the inclusion of the item. More sophisticated analyses based on modern test theory could be carried out in future to offer further insight into the LMUP's performance.

We recommend that item 1 (contraception) is kept under review as it showed low variability in endorsement in the main, classical test theory analysis, and narrowly missed selection using the Mokken scaling procedure (modern test theory analysis). A recent evaluation of the LMUP in India also found that item 1 showed little variability in endorsement and contributed little to the measure. It is possible that the item could be improved by revision of its response options. For instance, from the original UK development and evaluation study, we know that item 1 was understood almost exclusively in terms of artificial/modern methods of contraception, and incorporation of non-modern methods in the response options might be a way forward, a suggestion also made by Rocca et al. [33]. Alternatively, as evidence accumulates from the evaluation of the LMUP in different countries it may become apparent that item 1 would be better removed. At the moment, its inclusion does no great harm as the measure is still valid and reliable with its inclusion.

This validation of the LMUP measure in a U.S. population provides a contemporary, psychometrically-validated outcome measure of unplanned pregnancy which can be added to the U.S. toolkit of pregnancy planning measures. This will be critical for studies on contraception or abortion as well as to control for unplanned pregnancy in studies on antenatal care.
